# Differential mRNA expression of seven genes involved in cholesterol metabolism and transport in the liver of atherosclerosis-susceptible and -resistant Japanese quail strains

**DOI:** 10.1186/1297-9686-44-20

**Published:** 2012-06-08

**Authors:** Patricia Schulte, David V Godin, Kimberly M Cheng

**Affiliations:** 1 Avian Research Centre, Faculty of Land and Food Systems, The University of British Columbia, Vancouver, BC, Canada; 2 Department of Zoology, Faculty of Science, The University of British Columbia, Vancouver, BC, Canada; 3 Department of Anesthesiology, Pharmacology and Therapeutics, Faculty of Medicine, The University of British Columbia, Vancouver, BC, Canada; 4 Novozymes (China) Investment Co. Ltd., 14 Xinxi Road, Shangdi Zone, Haidian District, Beijing, China

## Abstract

**Background:**

Two atherosclerosis-susceptible and -resistant Japanese quail (*Coturnix japonica*) strains obtained by divergent selection are commonly used as models to study atherosclerosis, but no genetic characterization of their phenotypic differences has been reported so far. Our objective was to examine possible differences in the expression of genes involved in cholesterol metabolism and transport in the liver between these two strains and to evaluate the value of this model to analyze the gene system affecting cholesterol metabolism and transport.

**Methods:**

A factorial study with both strains (atherosclerosis-susceptible versus atherosclerosis-resistant) and two diets (control versus cholesterol) was carried out. The mRNA concentrations of four genes involved in cholesterol biosynthesis (*HMGCR*, *FDFT1*, *SQLE* and *DHCR7*) and three genes in cholesterol transport (*ABCG5*, *ABCG8* and *APOA1*) were assayed using real-time quantitative PCR. Plasma lipids were also assayed.

**Results:**

Expression of *ABCG5* (control diet) and *ABCG8* (regardless of dietary treatment) and expression of *HMGCR*, *FDFT1* and *SQLE* (regardless of dietary treatment) were significantly higher in the atherosclerosis-resistant than in the atherosclerosis-susceptible strain. Plasma triglyceride and LDL levels, and LDL/HDL ratio were significantly higher in the atherosclerosis-susceptible than in the atherosclerosis-resistant strain fed the cholesterol diet. In the atherosclerosis-susceptible strain, *ABCG5* expression regressed significantly and positively on plasma LDL level, whereas *DHCR7* and *SQLE* expression regressed significantly and negatively on plasma triglyceride level.

**Conclusions:**

Our results provide support for the hypothesis that the atherosclerosis-resistant strain metabolizes and excretes cholesterol faster than the atherosclerosis-susceptible strain. We have also demonstrated that these quail strains are a useful model to study cholesterol metabolism and transport in relation with atherosclerosis.

## Background

Atherosclerosis is a complex pathological process that is affected by both environmental and genetic factors; it is a major cause of morbidity and mortality in industrialized societies [1,2]. Although surgical and medical treatments have progressed, current therapies that slow the formation of atherosclerotic plaques are not totally successful [2]. Therefore, it is necessary to continue investigating the fundamental mechanisms that cause atherosclerosis to develop more effective forms of treatment e.g. [3].

Japanese quail (*Coturnix japonica*) was first used as a research model for atherosclerosis in the early 1960s [4], and since then, numerous studies have demonstrated the value of this model to obtain information on the development of hypercholesterolemia and atherosclerosis in man. One reason why the Japanese quail is a good model to study atherosclerosis is that it can develop “complex” vascular lesions (focal haemorrhage, calcification and fibrosis) that are very similar to lesions in man [5-7]. Divergent selection of Japanese quail for susceptibility and resistance to atherosclerotic plaque formation induced by dietary cholesterol have resulted in two strains i.e. atherosclerosis-susceptible (SUS) and atherosclerosis-resistant (RES) strains that are valuable models. The female Japanese quail does not develop atherosclerotic plaques even when exposed to a 0.5% w/w cholesterol diet [7]. Before selection, 8% of the males from a random-bred foundation population developed atherosclerosis when fed a high cholesterol diet (0.5% w/w) [5]. After divergent selection during four generations, 80% of the SUS males as compared to only 4% of the RES males developed atherosclerosis [5]. However, apart from the characterization of certain physiological differences between these two strains, no molecular characterization of the phenotypic differences has been carried out. Previous studies [5,8] have shown that after cholesterol feeding, plasma cholesterol levels remain high for a significantly longer time in the SUS than in the RES males. In addition, SUS males have fatty livers and higher amounts of liver cholesterol than RES males. Shih et al. [5] hypothesized that the RES individuals were more resistant because “they metabolized and excreted cholesterol faster than the SUS”. Therefore, in our study, we have compared the expression of several genes involved in cholesterol metabolism and transport in the liver of SUS and RES males.

The mevalonate pathway (or HMG-CoA reductase pathway) is an important component of the endogenous cholesterol biosynthesis pathway [9] in the liver. During the process of converting mevalonate to cholesterol and other sterol isoprenoids, many important enzymes such as 3-hydroxy-3-methylglutaryl-coenzyme A reductase (HMGCR or HMG-CoA reductase), squalene synthase (FDFT1), squalene expoxidase (SQLE), mevalonate kinase (MVK), phosphomevalonate kinase (PMVK) and 7-dehydrocholesterol reductase (DHCR7) are involved in regulating the overall process [9]. We examined the individual expression of the following genes *HMGCR*, *FDFT1*, *SQLE* and *DHCR7* by quantifying their mRNA levels in liver cells of SUS and RES males.

ABCG5 and ABCG8, from the ATP binding cassette (ABC) transporter family, are cholesterol excretion transporters [3]. Apolipoprotein A1 (APOA1) is the major protein component of HDL in plasma and is believed to have a protective effect against atherosclerosis by participating in the reverse transport of hepatic cholesterol from tissues to the bile for excretion [10-12]. Therefore, we also included the genes of these three proteins in our study.

## Methods

### Experimental birds

The SUS and RES quail stains were acquired by the University of British Columbia (UBC) Quail Genetic Resource Centre from the North Carolina State University in 1989. Since then, they have undergone further divergent selection for susceptibility and resistance to atherosclerotic plaque formation induced by dietary cholesterol (0.5%w/w) (KM Cheng, unpublished data).

### Experimental design

After hatching, both SUS (N = 50) and RES (N = 50) males were fed a semi-synthetic diet (Table 1) prepared by the feed mill at the Agriculture and Agri-Food Canada Research Station at Agassiz, British Columbia, according to the NRC nutrient requirement standards recommended for quail (http://www.nap.edu/catalog/2114.html). At six weeks of age, 13 birds (6 SUS and 7 RES) were euthanized and liver tissues were collected. The remaining birds were divided into two dietary treatment groups, fed either a regular synthetic diet or a synthetic diet containing cholesterol (0.5% w/w) (Table 1) for another six weeks (Table 2). At twelve weeks of age, 24 birds from each dietary treatment group were euthanized and liver tissue samples were collected for further analysis. This research was carried out with the approval of the UBC Animal Care Committee, Certificate # A06-1473.

**Table 1:** Semi-synthetic diets

**Ingredients (g/kg)**	**Control diet**	**Cholesterol diet**
Soy protein flour (50% protein)	340	340
Corn starch	400	390
Limestone	50	50
Mineral premix	5	5
Monofos	30	30
Sucrose	20	20
Alphacel	70	70
Vitamin premix	5	5
D-L methionine	4	4
Choline chloride	3.8	3.8
Tallow	50	50
Vegetable oil	30	30
Cholesterol	0	5
Cholic acid	0	2.5

**Table 2:** Dietary treatments (between weeks 7 and 12) and number of birds analyzed

**Diet treatments**	**SUS**	**RES**
Regular diet	6 males	6 males
Cholesterol diet (0.5% w/w)	6 males	6 males

### Preparation of total RNA and synthesis of first-strand cDNA

The birds were euthanized by decapitation. Livers were then quickly removed, dissected and stored in RNAlater reagent (Qiagen, Valencia, CA, USA) at −20°C until use. Total RNA from liver cells was extracted using RNeasy mini columns (Qiagen, Valencia, CA, USA). Concentration and purity were checked by spectrophotometer.

cDNA was synthesized using SuperScript™ ІІІ RT (200 units/μl) (Invitrogen Corporation, Carlsbad, CA, USA) at 50°C using Oligo (dT)_18_ primers (Fermentas Inc., Glen Burnie, MD, USA) according to the manufacturer's instructions. Each 38 μl reaction volume contained 5 μg of total RNA, 1 μl Oligo (dT)_18_ primers (100 mM), 2 μl dNTP (10 mM) (Fermentas Inc., Glen Burnie, MD, USA), 8 μl 5x first strand buffer, 4 μl DTT (0.1 M) and 2 μl SuperScript™ ІІІ RT. One μl of RiboLock™ RNase Inhibitor (40 U/μl) (Fermentas Inc., Glen Burnie, MD, USA) was added to each reaction mixture in order to inhibit RNA degradation during reverse transcription. The first-strand cDNA was stored at −20° C for future real-time PCR.

### Primer design

Primer pairs for each gene selected were designed using either Japanese quail (*Coturnix japonica*) or chicken (*Gallus gallus*) sequence information from the National Center for Biotechnology Information (NCBI, http://www.ncbi.nlm.nih.gov) GenBank database. The *glyceraldehyde 3-phosphate dehydrogenase* (*GAPDH*) gene was used as an internal control [13]. Real-time PCR primers were designed using Primer Express version 2.0.0 (Applied Biosystems, Foster City, CA, USA) and were ordered from IDT (Integrated DNA technologies, Coralville, IA, USA). Primer information is shown in Table 3.

**Table 3:** Real-time PCR primer combinations

**Primer name**	**Gene ID**	**Species**	**Primer**	**Primer sequence (5’- 3’)**
GAPDH	Z19086	*C. coturnix*	Forward	GGCACTGTCAAGGCTGAGAAT
			Reverse	GCATCTCCCCACTTGATGTTG
HMGCR	NM_204485	*G. gallus*	Forward	GCAGAGGGCCTTACAAC
			Reverse	GGAGGAGCAAGCCGTAT
FDFT1	NM_001039294	*G. gallus*	Forward	GCCATCATGTACCAGTATGTG GAA
			Reverse	GCTGCGTCTTGTTGGAGGAA
SQLE	NM_001030953	*G. gallus*	Forward	GAGGTAGAAATTCCTTTTCCAACATCT
			Reverse	GCCGTGATGGAAGGACCTT
DHCR7	XM_420914	*G. gallus*	Forward	GGGAAAGATTGGAAACGCTACA
			Reverse	CAGATTCTGTGTCAGCCTTAAAACA
ABCG5	XM_419457	*G. gallus*	Forward	ATTACAAGATCCCAAGGTCATGCT
			Reverse	GAGACGATCTGGTTTGCAGTCA
ABCG8	XM_419458	*G. gallus*	Forward	GCCTTCCAGCATGTTTTTCAG
			Reverse	CGCAACCGTAGCTCTGCTATT
APOA1	D85133	*C. coturnix*	Forward	TCTGGTGCAGGAATTCAAGGA
			Reverse	TCATCCAGGAGGTCGATCAAG

### Real-time PCR

An aliquot of the purified first strand cDNA templates was used to prepare the standard curve cDNA template mixture (calibrated sample), while the remainder was diluted to half the concentration for the real-time PCR. Real-time PCR was performed using an ABI Prism 7000 (Applied Biosystems, Foster City, CA, USA). Each sample was run in duplicate. The PCR was carried out in a reaction volume of 22 μl, containing 2 μl cDNA template (diluted 1:1 in water), 0.4 μl forward primer (10 μM), 0.4 μl reverse primer (10 μM) (Integrated DNA technologies, Coralville, IA) and 10 μl SYBR Green universal PCR Master Mix (Applied Biosystems, Foster City, CA, USA), and water was added to a final volume of 22 μl. The following PCR conditions were applied: 50° C for 2 min, 95° C for 10 min, and 40 cycles of denaturation at 95° C for 15 s and annealing and extension at 60° C for 1 min. Fluorescence measurements were recorded using SYBR as the reporter dye and the results were normalized to the endogenous control, *GAPDH*. A standard curve was produced for all primers using serial dilutions of cDNA (2x, 1x, 1/2x, 1/4x and 1/8x). The 2x mixture was prepared with the first-strand cDNA products (which had twice the concentration of template cDNA). Raw data analyses were done with the 7000 System Software (Applied Biosystems, Foster City, CA, USA). Expression levels were quantified by comparing the results of each real-time PCR to the standard curve produced by serial dilutions. Normalized mRNA levels were then calculated as the ratio of the measured amount of target gene mRNA to the amount of *GAPDH* mRNA.

### Plasma lipid assays

Plasma samples (N = 56; including samples from birds used for the real-time PCR analysis) from 6-week old (6 SUS/control and 7 RES/control) and 12-week old (16 SUS/control, 20 SUS/cholesterol, 7 RES/control, 6 RES/cholesterol) SUS and RES males fed both dietary treatments were sent to the Department of Pathology and Laboratory Medicine at St. Paul’s Hospital (Vancouver, BC) and assayed for total cholesterol, HDL, and triglycerides using enzymatic methods on an ADVIA 1650 Chemistry System. HDL was assessed by the direct method without precipitation of apolipoprotein B [14-16]. LDL values were determined by Friedewald’s formula, using measured values for total cholesterol, HDL and triglycerides [17,18].

### Statistical analysis

Least squares analysis of variance was performed using JMP 8.0 (SAS Institute, North Carolina, 2008). The statistical model for mRNA levels was:

(1) $${\text{Y}}_{\text{ijkl}}=\mu +{\text{ S}}_{\text{i}}+{\text{ D}}_{\text{j}}+{\text{ A}}_{\text{k}}+{\left(\text{SD}\right)}_{\text{ij}}+{\left(\text{SA}\right)}_{\text{ik}}+{\text{ E}}_{\text{ijkl}}$$

where Y_ijkl_ is the measure for the l^th^ individual of the i^th^ strain, j^th^ diet from k^th^ age group; S_i_ indicates whether the bird was RES or SUS; A_k_ represents the two age groups i.e. 6-week or 12-week old; D_j_ indicates whether the bird was on a regular diet or a cholesterol diet; (SD)_ij_ and (SA)_ik_ are interaction terms; and E_ijkl_ is the error term. The data were log-transformed before analysis. The results were reported as the least square mean values for each dataset ± standard error of means (SEM) and the level of statistical significance was defined at P < 0.05. Tukey’s HSD was used for mean separation.

For plasma lipid parameters, the following model was used:

(2) $${\text{Y}}_{\text{ijk}}=\mu +{\text{ S}}_{\text{i}}+{\text{ D}}_{\text{j}}+{\left(\text{SD}\right)}_{\text{ij}}+{\text{ E}}_{\text{ijk}}$$

The mRNA levels of the seven candidate genes were also regressed on the plasma lipid parameters with multiple regression analysis (JMP 8.0).

## Results

The effects of strain and diet on the mRNA expression of the seven genes examined are shown in Table 4. Because none of the 6-week old birds were fed the cholesterol-enhanced diet, we could examine the effects of strain and age on gene expression only for the birds fed the regular diet (Table 5).

**Table 4:** **Liver mRNA expressions**^**§**^**in SUS and RES quail fed regular or cholesterol diets**

**Gene**	**Effect**	**Regular diet**		**Cholesterol diet**	
		**RES (N = 13)**	**SUS (N = 12)**	**RES (N = 6)**	**SUS (N = 6)**
*HMGCR*	*P < 0.01	1.059 ± 0.080	0.776 ± 0.080	1.082 ± 0.127	0.946 ± 0.127
*FDFT1*	*P < 0.04; †P < 0.02	1.220 ± 0.125	0.598 ± 0.125	0.563 ± 0.177	0.529 ± 0.177
*DHCR7*		1.209 ± 0.157	1.094 ± 0.157	1.012 ± 0.222a	0.673 ± 0.222b
*SQLE*	†P < 0.01	1.782 ± 0.308	0.392 ± 0.320	0.044 ± 0.453	0.008 ± 0.453
*ABCG5*	*† P < 0.05	0.914 ± 0.088a	0.566 ± 0.092b	0.959 ± 0.130ab	1.064 ± 0.130a
*ABCG8*	*P < 0.01; †P < 0.001	1.153 ± 0.163	0.609 ± 0.169	1.856 ± 0.240	1.331 ± 0.240
*APAO1*	†P < 0.001	0.857 ± 0.097	0.785 ± 0.101	1.382 ± 0.143	1.412 ± 0.143

N = number of individuals measured; total number of individuals = 37; *denotes significant strain effect; †denotes significant diet effect; *†denotes significant strain x diet interaction; ^§^all values (± SEM) indicate the gene of interest relative to *GAPDH* (arbitrary units).

**Table 5:** **- Liver mRNA expressions**^**§**^**in 6-week old and 12-week old SUS and RES quail fed the regular diet**

**Gene**	**Effect**	**6-week old**		**12-week old**	
		**RES (N = 7)**	**SUS (N = 6)**	**RES (N = 6)**	**SUS (N = 6)**
*HMGCR*	*P < 0.02	1.110 ± 0.110	0.688 ± 0.110	1.009 ± 0.110	0.864 ± 0.110
*FDFT1*	*P < 0.01	1.359 ± 0.217	0.558 ± 0.217	1.082 ± 0.217	0.638 ± 0.217
*DHCR7*	†P < 0.01	1.423 ± 0.250	1.359 ± 0.250	0.995 ± 0.250	0.829 ± 0.250
*SQLE*	*P < 0.02	2.430 ± 0.483	0.414 ± 0.522	1.026 ± 0.522	0.370 ± 0.522
*ABCG5*	*†P < 0.0005	0.594 ± 0.076b	0.547 ± 0.082b	1.288 ± 0.082a	0.586 ± 0.082b
*ABCG8*	*P < 0.01; † P < 0.01	0.724 ± 0.189	0.531 ± 0.204	1.654 ± 0.204	0.687 ± 0.204
*APAO1*	†P < 0.0001	0.626 ± 0.093	0.448 ± 0.101	1.126 ± 0.101	1.123 ± 0.101

N = number of individuals measured; total number of individuals = 25; *denotes significant strain effect; †denotes significant age effect; *†denotes significant strain x age interaction; ^§^all values (± SEM) indicate the gene of interest relative to GAPDH (arbitrary units).

### Mevalonate pathway genes

#### HMGCR

The expression of *HMGCR* was significantly (P < 0.01) higher in RES (1.08 ± 0.07 HMGCR/GAPDH) than in SUS birds (0.82 ± 0.07 HMGCR/GAPDH), regardless of dietary treatment and age (Table 4). No significant interaction effect was detected.

#### FDFT1

The expression of *FDFT1* was significantly (P < 0.04) higher in RES (0.89 ± 0.11 FDFT1/GAPDH) than in SUS birds (0.56 ± 0.11 FDFT1/GAPDH), regardless of dietary treatment and age (Table 4). No significant interaction effect was detected.

#### SQLE

The expression of *SQLE* was significantly (P < 0.02) higher in RES than in SUS individuals when the birds were fed the regular diet (Table 5), and its expression in both strains was significantly (P < 0.01) suppressed when the birds were fed the cholesterol diet (Table 4). However, the strain x diet interaction was not significant (P = 0.51).

#### DHCR7

There were no strain differences (P = 0.35) in the expression of *DHCR7* when the birds were fed the control diet (Table 5). When challenged by the cholesterol diet, its expression was significantly (P < 0.04) higher in RES (1.012 ± 0.01 DHCR7/GAPDH) than in SUS birds (0.673 ± 0.01 DHCR7/GAPDH) (Table 4). However, the strain x diet interaction was not significant (P = 0.6). Six-week old birds (1.36 ± 0.18 DHCR7/GAPDH) had a significantly (P < 0.01) higher *DHCR7* expression than 12-week old birds (0.88 ± 0.10 DHCR7/GAPDH) (Table 5).

### ATP-cassette binding transporter genes

#### ABCG5

There was a significant (P < 0.05) diet x strain interaction in *ABCG5* gene expression. When fed the regular diet, expression of *ABGC5* was significantly higher in RES than in SUS birds. When challenged with the cholesterol diet, the difference in expression level in the two strains became non-significant (Table 4). There was a significant (P < 0.0005) strain x age interaction for birds on the regular diet (Table 5); the expression of *ABCG5* significantly increased with age in RES birds but not in SUS birds.

#### ABCG8

The expression of *ABCG8* was significantly (P < 0.02) higher in RES (1.50 ± 0.14 ABCG8/GAPDH) than in SUS birds (0.97 ± 0.15 ABCG8/GAPDH) (Table 4). Birds fed the cholesterol diet (1.59 ± 0.17 ABCG8/GAPDH) had significantly (P < 0.002) higher *ABCG8* expression than birds on the control diet (0.88 ± 0.12 ABCG8/GAPDH) (Table 4). Twelve-week old birds (1.38 ± 0.11 ABCG8/GAPDH) on regular diet had a significantly (P < 0.004) higher *ABCG8* expression than 6-week old birds (0.82 ± 0.19 ABCG8/GAPDH) on the same diet (Table 5).

#### APOA1

There was no significant (P = 0.46) strain effect on the expression of *APOA1*. Both RES and SUS birds fed the cholesterol diet (1.40 ± 0.06 APOA1/GAPDH) had a significantly (P < 0.001) higher expression compared to birds fed the regular diet (0.82 ± 0.05 APOA1/GAPDH) (Table 4). Twelve-week old birds (1.12 ± 0.07 APOA1/GAPDH) on regular diet had a significantly (P < 0.0001) higher *APOA1* expression than 6-week old birds (0.54 ± 0.07 APOA1/GAPDH) on the same diet (Table 5).

### Plasma total cholesterol and LDL levels

There was a significant strain x diet interaction for plasma total cholesterol (P < 0.013) and LDL (P < 0.01) levels. Plasma total cholesterol and LDL levels in SUS and RES birds fed the regular diet were not different but they were significantly (P < 0.0001) higher when the birds were fed the cholesterol diet, with levels in SUS birds significantly (P < 0.05) higher than those in RES birds (Tables 6 and 7).

**Table 6:** Plasma total cholesterol levels* and triglyceride levels** in SUS and RES quail fed regular or cholesterol diets

**Diet**	**SUS**		**RES**	
	**Total cholesterol (mmol/L)**	**Triglycerides (mmol/L)**	**Total cholesterol (mmol/L)**	**Triglycerides (mmol/L)**
Regular	7.45 ± 2.58 c	0.98 ± 0.32 B	6.15 ± 3.90 c	1.18 ± 0.48 B
Cholesterol	42.43 ± 1.98 a	3.32 ± 0.24 A	24.11 ± 4.21 b	1.54 ± 0.52 B

Total number of individuals measured N = 56; P < 0.01 and P < 0.02, respectively *total cholesterol means followed by different lower case letters are significantly different by Tukey’s HSD; **plasma triglycerides means followed by different capital letters are significantly different by Tukey’s HSD.

**Table 7:** Plasma LDL levels* (N = 51; P < 0.01) and HDL levels)** (N = 56; P < 0.028) in SUS and RES quail fed regular or cholesterol diets

**Diet**	**SUS**		**RES**	
	**LDL (mmol/L)**	**HDL (mmol/L)**	**LDL (mmol/L)**	**HDL (mmol/L)**
Regular	1.75 ± 2.03 c	5.25 ± 0.21 A	1.64 ± 3.07 c	3.96 ± 0.32 B
Cholesterol	32.82 ± 1.73 a	4.81 ± 0.16 AB	18.64 ± 3.32 b	4.75 ± 0.35 B

*plasma LDL means followed by different lower case letters are significantly different by Tukey’s HSD; **plasma HDL means followed by different capital letters are significantly different by Tukey’s HSD.

### Plasma triglyceride levels

There was a significant (P < 0.02) strain x diet interaction in plasma triglyceride levels. Plasma triglyceride levels in SUS and RES birds fed the regular diet were not different. When the birds were fed the cholesterol diet, plasma triglyceride levels increased significantly (P < 0.05) in SUS but not in the RES birds (Table 6).

### Plasma HDL levels and LDL/HDL ratio

There was a significant (P < 0.028) strain x diet interaction in plasma HDL levels. Plasma HDL levels were significantly (P < 0.05) higher in SUS than in RES birds when they were on the regular diet. Plasma HDL levels did not change significantly when the birds were fed the cholesterol diet; however, the difference between the SUS and RES birds became non-significant (Table 7). These small changes in the HDL level did not affect the LDL/HDL ratio (see Table 8 and Table 7).

**Table 8:** Plasma LDL/HDL ratio in SUS and RES quail fed regular or high cholesterol diets (N = 51; P < 0.0042)

**Diet**	**SUS**	**RES**
Regular	0.34 ± 0.43 c	0.42 ± 0.66 c
Cholesterol	7.16 ± 0.37 a	3.84 ± 0.71 b

*means followed by different letters are significantly different by Tukey’s HSD.

### Regression of gene expression on plasma lipid levels

**Figure 1:**
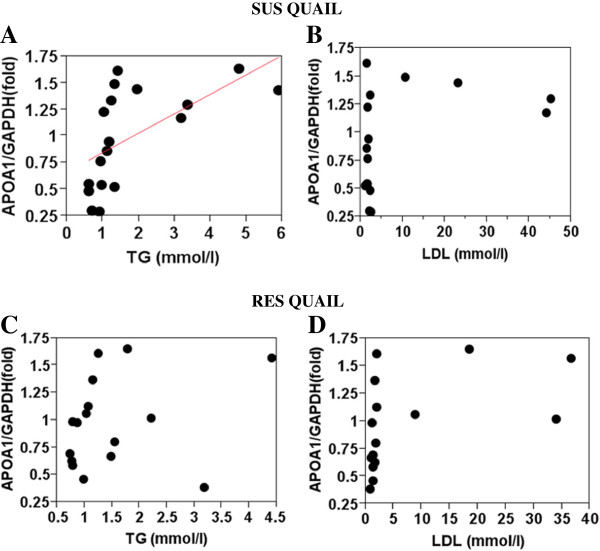
***APOA1*expression in SUS and RES liver relative to plasma triglyceride and LDL levels.** In part A: r^2^ = +0.36; P < 0.009.

**Figure 2:**
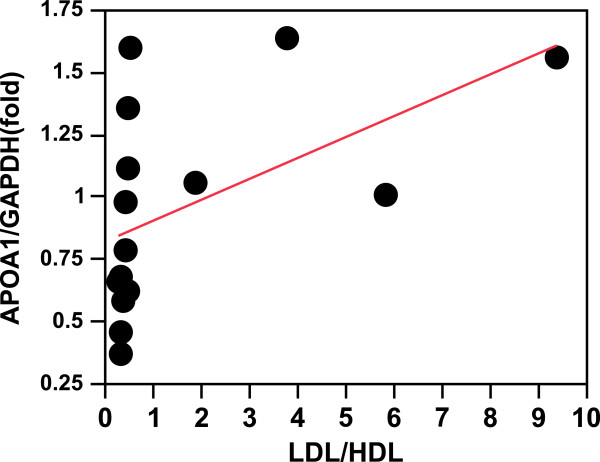
***APOA1*expression in RES liver relative to LDL/HDL ratio.** r^2^ = 0.28; P < 0.04.

Because regulation of gene expression may differ between the two strains, they were analysed separately (Table 9). *APOA1* expression regressed (r^2^ = 0.36) significantly (P < 0.009) on plasma triglyceride level in the SUS but not in the RES birds (Table 9, Figure 1A and C). In RES birds, *APOA1* expression tended (P < 0.06; r^2^ = 0.24) to regress on LDL (Table 9, Figure 1D) and regressed significantly (P < 0.04; r^2^ = 0.28) on the LDL/HDL ratio (Figure 2).

**Table 9:** Regression of gene expression on plasma lipid levels

mRNA expression*		**SUS**	**RES**
HMGCR	Plasma triglyceride	P = 0.60 NS	P = 0.94 NS
	LDL	P = 0.30 NS	P = 0.39 NS
FDFT1	Plasma triglyceride	P = 0.99 NS	P = 0.26 NS
	LDL	P = 0.71 NS	P = 0.12 NS
DHCR7	Plasma triglyceride	r^2^ = −0.23; P < 0.04	P = 0.80 NS
	LDL	P = 0.29 NS	P = 0.63 NS
SQLE	Plasma triglyceride	r^2^ = −0.29; P < 0.02	P = 0.09 NS
	LDL	r^2^ = −0.25; P < 0.04	P = 0.16 NS
ABCG5	Plasma triglyceride	P = 0.11 NS	P = 0.47 NS
	LDL	r^2^ = +0.48; P < 0.008	P = 0.84 NS

*There was no significant regression of mRNA expression on HDL levels; *NS* = not significant.

**Figure 3:**
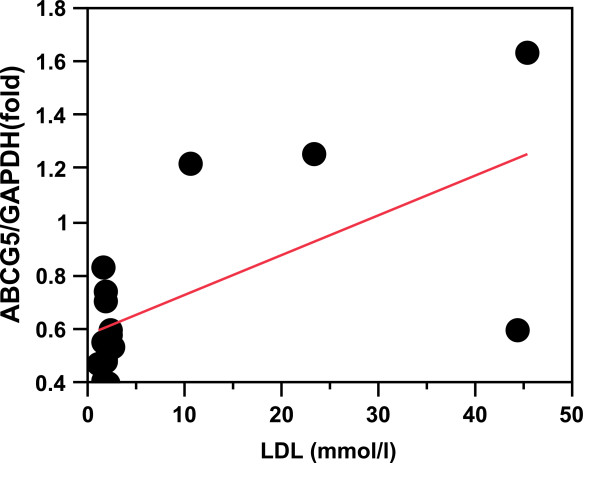
***ABCG5*expression in SUS liver relative to plasma LDL levels.** r^2^ = 0.48; P < 0.008.

**Figure 4:**
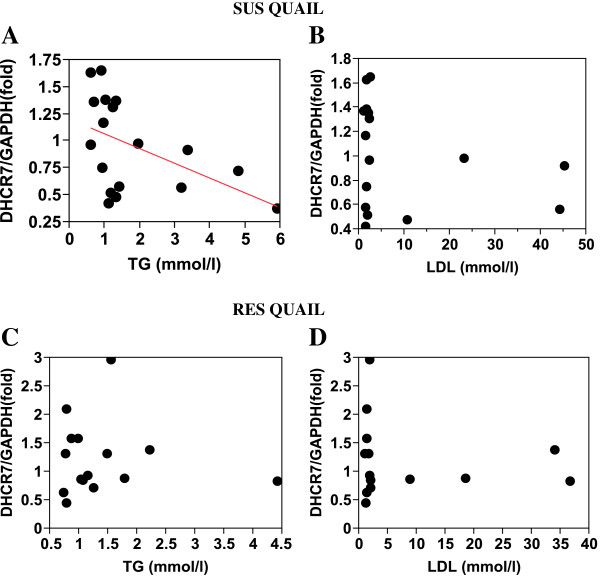
***DHCR7*expression in SUS and RES liver relative to to plasma triglyceride and LDL levels.** In part A: r^2^ = −0.23; P < 0.04.

**Figure 5:**
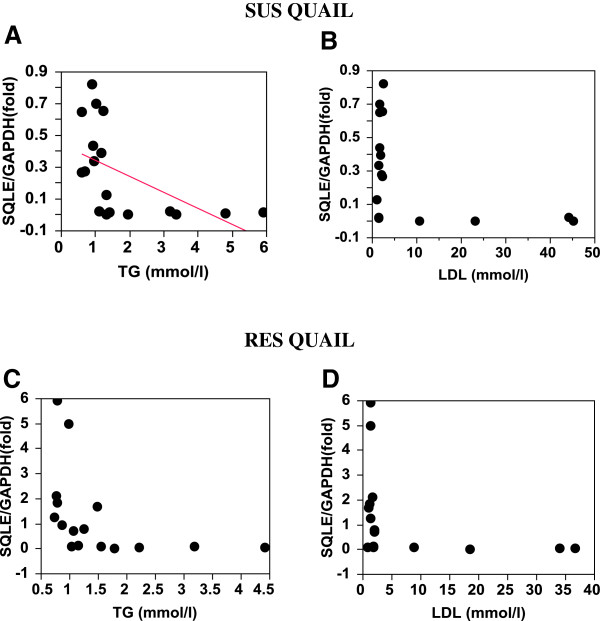
***SQLE*expression in SUS and RES liver relative to plasma triglyceride and LDL levels.** In part A: r^2^ = −0.29; P < 0.02.

In the SUS birds, *ABCG5* expression regressed (r^2^ = 0.48) significantly (P < 0.008) and positively on plasma LDL level (Table 9, Figure 3), whereas *DHCR7* and *SQLE* expression regressed significantly (P < 0.04 and P < 0.02, respectively) and negatively on plasma triglyceride level (Table 9, Figures 4A and 5A).

**Figure 6:**
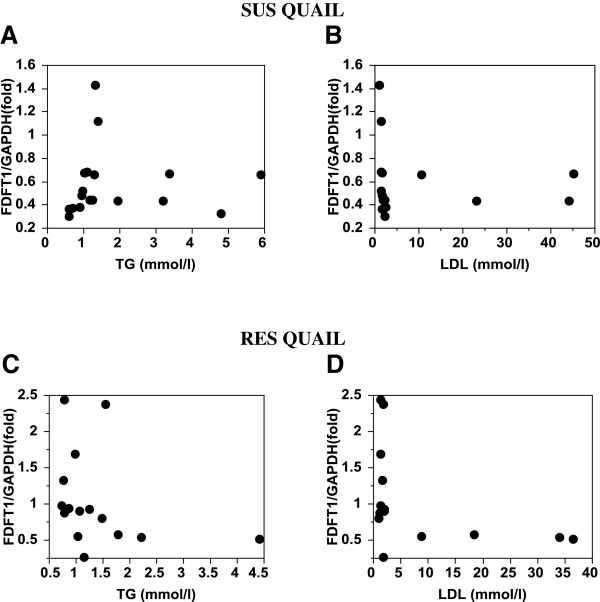
***FDFT1*expression in SUS and RES liver relative to plasma triglyceride and LDL levels.**

No other significant regression of gene expression on plasma lipid levels was found in the RES birds. However, given the fact that only a few individuals showed high levels of plasma lipid, a linear relation between gene expression and plasma lipid levels may not be valid. Examination of the regression plots revealed that in both the SUS and RES birds, *SQLE* and *FDFT1* expressions were completely or drastically suppressed when plasma triglycerides or LDL reached a threshold level (Figures 5 and 6). *DHCR7* expression appears to follow the same pattern (Figure 4).

## Discussion

The RES and SUS quail strains have been developed through divergent selective breeding from the same foundation population [5], and thus should be genetically similar except for the changes induced by selection. Previously, it has been reported that one of the observable differences between RES and SUS individuals is that after being fed on a cholesterol-enhanced diet, plasma cholesterol levels remain elevated in SUS individuals significantly longer than in RES individuals [5]. Since the liver plays a key role in regulating cholesterol homeostasis by acting as the main site for lipid metabolism and bile salt formation, we have focused our study on this organ and more specifically on the expression in liver of several cholesterol biosynthesis and transporting genes. Although gene expression is a phenotype and not a genotype, it probably reflects more directly genotypic changes than morphological or physiological phenotypes.

### Strain differences in gene expression

*ABCG8* expression was significantly higher in the liver of RES as compared to SUS individuals under all dietary conditions, while *ABCG5* expression was higher only under some dietary conditions. Evidence from both animal models and research on man supports the important role of these two ABC transporters in the regulation of the excretion of sterols from the liver via bile to prevent the accumulation of dietary sterols [19]. In human, mutations in either of these genes cause sitosterolemia, a disorder that is characterized by intestinal hyper-absorption of all sterols and impaired ability to excrete sterols into bile. Patients develop tendon and tuberous xanthomas, accelerated atherosclerosis, and premature coronary artery disease [19-21]. Sitosterolemia is caused by an abnormal expression pattern of the ABC transporters (heterodimers of sterolin-1 and sterolin-2), which function as gatekeepers for dietary sterol uptake and excretion [19]. A point mutation in exon 8 of the *ABCG5* gene causes premature termination of translation resulting in a truncated and non-functional sterolin-1 protein. It has also been reported that several mutations in *ABCG8* result in a truncated non-functional protein [19,22]. Besides, another point mutation in the human *ABCG5* gene enhancing the ABCG5/8 pathway has been shown to protect against atherosclerosis by increasing cholesterol elimination in the bile and reducing plasma cholesterol levels [23]. Furthermore, a study on the mouse has shown that over-expression of *ABCG5* and *ABCG8* decreases diet-induced atherosclerosis, in association with reduced liver and plasma cholesterol levels [20]. Another study of a partially inbred strain of opossums (*Monodelphis domestica*) with low levels of *ABCG5* and *ABCG8* expression was associated with an elevation in diet-induced VLDL and LDL cholesterol [24]. In our study, the lower *ABCG8* and *ABCG5* expression in the SUS individuals may be at least partially responsible for the greater susceptibility of this strain to diet-induced atherosclerosis [24].

In the RES birds, *ABCG5* expression remained high regardless of dietary treatment, whereas in the SUS birds, an increased level of *ABCG5* expression could be induced by a cholesterol-enhanced diet. Selection for atherosclerosis susceptibility may have altered the regulation of the *ABCG5* gene in the RES strain. Studies on mice have provided evidence for the direct control of *ABCA1ABCG5*, and *ABCG8* mRNA expression by the liver X receptor (LXR) pathway. Indeed, in mice fed with a cholesterol-enhanced diet, *ABCA1ABCG5*, and *ABCG8* mRNA expressions were up-regulated [25,26]. Liver X receptors and retinoid X receptors (RXR) form RXR/LXR heterodimer transcription factors that act as intracellular sterol sensors [27]. Accordingly, selection for atherosclerosis resistance may not have altered the expression of *ABCG5* but rather the expression of some of these receptor genes [26,28,29] thus permitting expression of *ABCG5* to remain in an up-regulated state in the RES individuals. It would be interesting to examine the expression of the *LXR* and *RXR* genes in the two quail strains under similar and different dietary conditions.

While we have measured the levels of plasma cholesterol and triglycerides, we have not determined their intracellular levels. The *LDL receptor* gene is regulated by the sterol regulatory element-binding protein (SREBP) pathway via negative feedback [30-32]. When intracellular cholesterol levels are high, the *LDL receptor* gene is down-regulated [31]. With fewer receptors, the liver takes up the LDL from blood less efficiently, and as a result, plasma LDL levels increase. It is possible that selection has altered the expression of the *LDL receptor* gene in the SUS and RES strains. SUS individuals are less efficient in removing excess cholesterol from the liver, thus they may have a down-regulated expression of the *LDL receptor* gene resulting in a higher level of dietary cholesterol remaining in circulation. Therefore, it would be relevant to examine the expression of the *LDL receptor* gene in the two quail strains fed with different diets.

The expression of three genes involved in cholesterol biosynthesis, *HMGCR*, *FDFT1* and *SQLE*, was found lower in SUS than in RES birds, regardless of diet and age. As a counteraction effect to the selective pressure for susceptibility to atherosclerosis, natural selection may have caused a permanent down-regulation of these genes in the SUS individuals to decrease endogenous cholesterol synthesis to maintain homeostasis. Apparently, this mechanism to maintain homeostasis became ineffective when the birds are fed a diet containing an extremely high level of cholesterol. These mevalonate pathway genes are also regulated by the intracellular cholesterol via the SREBP pathway [31]. For example, *HMGCR* has been shown to be regulated by sterol and non-sterol metabolites derived from mevalonate in a negative feedback loop [31,33,34]. Similar to *HMGCR*, *FDFT1* and *SQLE* are transcriptionally regulated via the SREBP pathway [25]. We have also found that the expression level of some of these genes regressed negatively on plasma LDL and triglyceride levels. A very high level of plasma cholesterol is associated with suppressed or strongly reduced expression of *FDFT1SQLE* and *DHCR7*. Although they may not be the primary rate-limiting enzymes in cholesterol biosynthesis [24], suppression of their expression may also be a protective action to turn off the endogenous cholesterol synthesis.

Thus, it is reasonable to hypothesize that the high intracellular cholesterol levels in the liver cells of the SUS birds may be related to the sub-normal functioning of the transporter genes *ABCG8* and *ABCG5*. The down-regulation of the mevalonate pathway genes may be an ineffective attempt to normalize intracellular cholesterol levels in the liver cells of the SUS birds.

## Conclusions

Cholesterol metabolism and transport are regulated by a complicated gene system. The number of genes that we have sampled in this study remains small and from a single tissue, thus we cannot draw any conclusion on how this gene system works or how this gene system has been affected by selective breeding. However, the SUS strain responded to selection in a short time (i.e. four generations) and then reached a plateau, which is an indication that only a few genes have been altered. Our results do provide some explanation for the plasma cholesterol levels remaining high for a significantly longer time in the SUS males than in the RES males. With the progress in micro-array technology and transcriptome pyrosequencing [35], this quail model will be useful to study the ramification effects of a few genes in the complicated gene system that affects atherosclerosis.

## Abbreviations

ABC, ATP binding cassette; ABCG5, ATP-binding cassette sub-family G member 5; ABCG8, ATP-binding cassette sub-family G member 8; ABCA1, member 1 of human transporter sub-family; ABCA, also known as cholesterol efflux regulatory protein; APOA1, apolipoprotein A1; BC, British Columbia; DHCR7, 7-dehydrocholesterol reductase; FDFT1, squalene synthase; GAPDH, Glyceraldehyde-3-phosphate dehydrogenase; HDL, high density lipoprotein; HMGCR, 3-hydroxy-3-methylglutaryl-coenzyme A reductase; LDL, low density lipoprotein; MVK, mevalonate kinase; NCBI, National Center for Biotechnology Information; NRC, National research council, USA; PMVK, phosphomevalonate kinase; RES, atherosclerosis-resistant quail strain; SUS, atherosclerosis-susceptible quail strain; SQLE, squalene expoxidase; UBC, University of British Columbia; w/w, weight/weight.

## Competing interests

The authors declare that they have no competing interests.

## Authors’ contributions

This manuscript is an extension of the thesis research carried out by Xinrui Li. David Godin provided expertise in cholesterol metabolism and atherosclerosis. Patricia Schulte provided expertise in gene expression analysis and the laboratory facility for carrying out the research. Kimberly Cheng contributed in writing and editing the manuscript. All authors read and approved the final manuscript.
